# Retentive Characteristics of a Polyetheretherketone Post-Core Restoration with Polyvinylsiloxane Attachments

**DOI:** 10.3390/polym12092005

**Published:** 2020-09-03

**Authors:** Ping Li, Dorina Hasselbeck, Alexey Unkovskiy, Feraydoon Sharghi, Sebastian Spintzyk

**Affiliations:** 1 Section Medical Materials Science and Technology, University Hospital Tübingen, Osianderstrasse 2-8, 72076 Tübingen, Germany; dorina.hasselbeck@googlemail.com (D.H.); sebastian.spintzyk@med.uni-tuebingen.de (S.S.); 2 Department of Prosthodontics, Geriartric Dentistry and Craniomandibular Disorders, Charité-Universitätsmedizin Berlin, Corporate Member of Freie Universität Berlin, Humboldt-Universität zu Berlin, and Berlin Institute of Health, Dental Materials and Biomaterial Research, Aßmannshauser Str. 4-6, 14197 Berlin, Germany; alexey.unkovskiy@charite.de; 3 Department of Dental Surgery, Sechenov First Moscow State Medical University, Trubetskaya Str. 8-2, 119991 Moscow, Russia; 4 Department of Prosthodontics, University Hospital Tübingen, Osianderstrasse 2-8, 72076 Tübingen, Germany; feraydoon.sharghi@med.uni-tuebingen.de

**Keywords:** polymer-based material, polyvinylsiloxane, polyetheretherketone, attachment system, overdenture, post-core restoration, prosthodontics, dental materials

## Abstract

A new application of a polyetheretherketone (PEEK) post-core restoration combined with polyvinylsiloxane (PVS) attachments was proposed and substantiated. This study aimed to evaluate retentive characteristics of the PEEK post-core restoration with PVS attachment systems. Specimens with PVS attachments were fabricated to evaluate retention force during 10,000 cyclic dislodgements. Additionally, the retention forces of PVS attachments with three different values of Shore hardness were further measured before and after aging treatments. The results of cyclic dislodgement test indicated a strong negative linear relationship between the cyclic times and retention force (*p* < 0.0001, *r* = −0.957). Furthermore, the retention forces of the PVS were significantly improved with Shore hardness increased, which was also affected by the aging treatment (F (2, 138) = 10.95, *p* < 0.0001). Therefore, the PEEK post-core restoration with PVS attachments exhibited the favorable retention force, which could be a promising alternative for dental prostheses.

## 1. Introduction

Edentulism and severe tooth loss affects hundreds of millions of people worldwide and compromises the normal mastication process, phonetics, and overall aesthetics [1]. Clinically, removable dental prostheses (RDPs) have been widely applied to replace the lost teeth and reestablish oral functions [2,3,4]. RDPs with attachment system exhibit superior clinical performance, due to improved stability (resistance to horizontal forces) and retention (resistance to vertical dislodgement) [5,6]. Nonetheless, attachment-retained RDPs demonstrated the highest rate of tooth fracture (12.7–40%), compared to clasp-retained RDPs and double crown-retained RDPs [6,7]. Considering endodontically treated teeth as an abutment, choosing materials of endodontic posts plays the predominant role to prevent the root fracture [8,9].

The ideal physical properties of post materials are required to be not only closer that of dentin, but also bonded to the tooth structure [10,11,12]. Traditional post-core restoration was fabricated from metallic materials, which have intrinsic rigidity that may increase the risk of root fracture [9,13]. By contrast, polymeric materials have been widely used for a broad prosthodontic application due to their excellent biocompatibility and appropriate mechanical properties [14,15]. Although the elastic modulus of fiber posts is closer to that of dentin, the fiber posts with low mechanical strength are susceptible to fracture itself, especially for RDPs anchoring [16,17]. Recently, polyetheretherketone (PEEK) has been recognized as a promising material for dental applications due to its low elastic modulus and high fracture resistance. The properties of PEEK may be considered as a valid material for post and core restorations [18,19,20].

The retentive characteristics of the attachment system represent a critical factor of clinical outcomes for attachment-retained RDPs or root supported overdenture. Conventional attachment systems have various types and provide unique versatility, mainly including stud-type attachments, ball-type attachments, magnetic attachments, and locator root attachments. However, these attachment systems still have been various drawbacks, such as low retention and stability, poor clinical manageability, high cost, etc. [21]. To date, polyvinylsiloxane (PVS) has been proposed and investigated as a matrix material for the attachment. Previous studies reported that PVS overdenture attachment exhibits the stability retention force and an economical chairside technique [22,23]. However, to the best of our knowledge, only a few studies focused on the selection of the attachment system with a PEEK post-core restoration.

**Figure 1:**
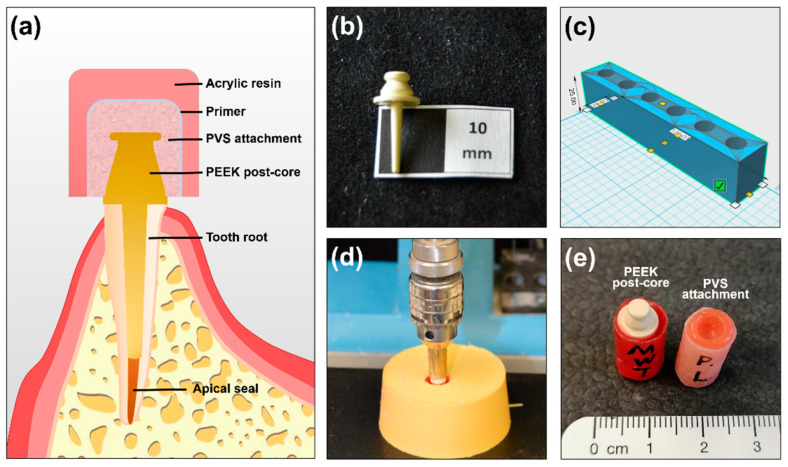
Experimental specimens used in the study. (**a**) Simplified schematic design of a novel polyetheretherketone (PEEK) post-core restoration with a polyvinylsiloxane (PVS) attachment system; (**b**) PEEK post-core restoration; (**c**) a holding device designed by computer-aided design; (**d**) insertion using a parallel guidance device; (**e**) tested specimens (left: a PEEK post-core restoration, right: a PVS attachment).

Based on the consideration above, the present study proposes a new clinical concept to apply a PEEK post-core restoration combined with a PVS attachment overdenture system (Figure 1a). This study aims to investigate the retention of these two materials relative to each other. The first null hypothesis was that there would be no statistically significant relationship between the retention force of the PVS attachment and long-term cyclic dislodgement. The second null hypothesis was that the retentive forces of the attachments would not be affected by the type of PVS attachments, irrespective of the aging treatment.

## 2. Materials and Methods

### 2.1. Specimen Preparation

Specimens of a PEEK post-core restoration were fabricated via injection molding technique (Wurzelstifte BioHPP, Bredent medical, Senden, Germany), as shown in Figure 1b. PEEK granular (BioHPP Granulat, Bredent, Senden, Germany) was used to fill into a metal mold at an injecting temperature of 400 °C. Furthermore, specimens with PVS attachment were further prepared to simulate an attachment system for RDPs. As depicted in Figure 1c, a customized cylindrical hollow mold with a diameter of 10 mm was designed by computer-aided design (CAD, 123D Design, Autodesk Inc., San Rafael, CA, USA) software. The holding mold was 3D printed using the fused deposition modelling (FDM) method (MakerBot, New York, NY, USA). Additionally, PEEK post-core restoration was fixed in the self-curing acrylic resin (Palavit G Liquid, Heraeus Kulzer, Hanau, Germany) and vertically inserted in the resin using a parallel guidance device (Figure 1d). Regarding the preparation of PVS attachments, denture base resin was filled into the mold, followed with cured according to the manufacturer’s instructions (Candulor Dental GmbH, Rielasingen-Worblingen, Germany). A homogenized hole was prepared on the denture base resin and then conditioned by a primer (Multisil, Bredent medical, Senden, Germany). The PVS attachments with three different degrees of Shore hardness were used as previously reported [22,23], denoted as SH25, SH50, and SH65, respectively (retention.sil −200, −400 and −600, Bredent medical, Senden, Germany), and cured followed by the manufacturer’s instructions (Figure 1e).

### 2.2. Cyclic Dislodgement Testing

**Figure 2:**
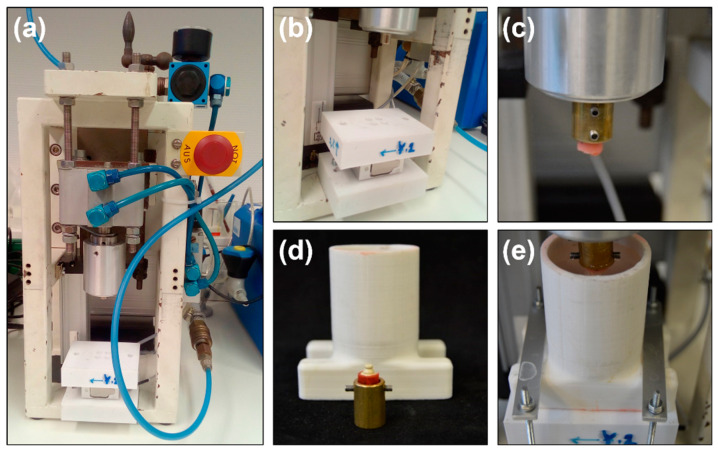
Experimental device for cyclic dislodgement testing. (**a**) Overview image of a Beisser machine; (**b**) working platform; (**c**) a metal holder with a specimen of PVS attachment; (**d**) a specimen of PEEK post-core restoration; (**e**) fixation of a specimen during the testing.

A total of ten specimens with SH65 attachment were conducted to investigate the effect of cyclic dislodgement on the retention force. A custom-made “Beisser” device was designed to simulate the repeated insertion–removal between a PEEK post-core restoration and PVS attachments in the long-term application, termed to cyclic dislodgement in this test. As shown in Figure 2a,b, the device was a pressure machine controlled by compressed air with a load sensor measuring the retention forces. The Beisser device was controlled by a GSV multichannel program (programmed by Mr Günter Wedenig from University Hospital Tübingen). In addition, the samples of PVS attachment were fixed with a metal holder (Figure 2c). The specimens of the PEEK post-core restoration were fixed and immersed in the artificial saliva in a container, as shown in Figure 2d. The artificial saliva was mainly composed of 0.21 g/L CaCl_2_·2H_2_O, 0.96 g/L Na_2_HPO_4_·2H_2_O, and 2.2 g/L mucins (Sigma, Germany), as reported before [24]. The cyclic dislodgement was set to 10,000 cycles at 0.67 cycles per second (Hz). The retention forces were recorded at every 500 cycles, which the built-in load cell absorbs forces of up to 50 N. (Figure 2e).

### 2.3. Retention Force Measurement

**Figure 3:**
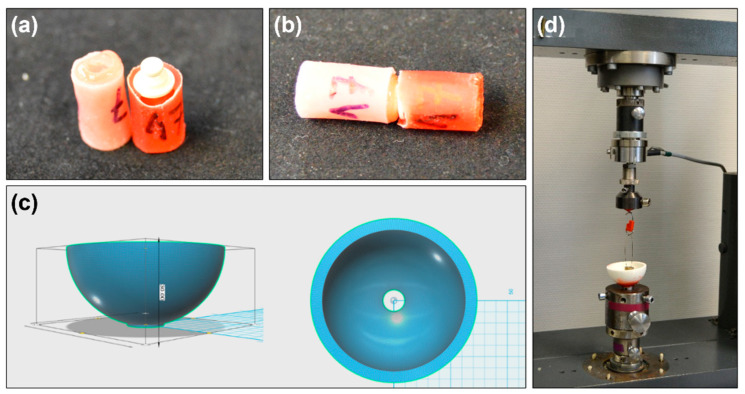
Experimental set-up of the universal testing machine. (**a**,**b**) Specimens of experimental samples after the aging treatment; (**c**) a container for artificial saliva designed by CAD; (**d**) the universal testing machine with an FDM 3D-printed container.

To further evaluate the effect of aging on the retention force changes, specimens of attachment (SH25, SH50, and SH65) and PEEK post-core restoration (*n* = 8 per groups) were used. As shown in Figure 3a,b, specimens were age treated through the mechanical dislodgements using a Beisser machine. For the aged specimens, the mechanical cyclic dislodgement was performed by 10,000 cycles at 0.67 cycles per second in artificial saliva, as the same with aforementioned (Section 2.2.). Next, a container for artificial saliva in the universal testing machine was designed in CAD software (reference) (Figure 3c). The specimens before and after aging were conducted to measure retention forces using a universal testing machine (Z010, Zwick GmbH, Ulm, Germany). Subsequently, each specimen fixed with a metal hold was immersed in the artificial saliva during the measurement (Figure 3d). The retention force (N) was measured with a 500 N load cell at a crosshead speed of 50 mm/min until complete separation, as reported before [25,26]. Measurements for each sample were repeated three times.

### 2.4. Statistical Analysis

All data were checked for normal distribution using the D’Agostino–Pearson omnibus normality test. The first working hypothesis was tested using a Pearson correlation coefficient (*R* value) and *p* values (two-tailed) between the retention force and cycle times. The second working hypothesis was analyzed using a two-way analysis of variance (ANOVA) with attachment system and aging as independent factors, followed by Tukey’s multiple comparisons test. Statistical analyses were performed by the software of GraphPad Prism (Prism 6.01, GraphPad Software, San Diego, CA, USA), and statistical significance was defined as *p* < 0.05.

## 3. Results

### 3.1. Cyclic Dislodgement of PVS Attachment

**Figure 4:**
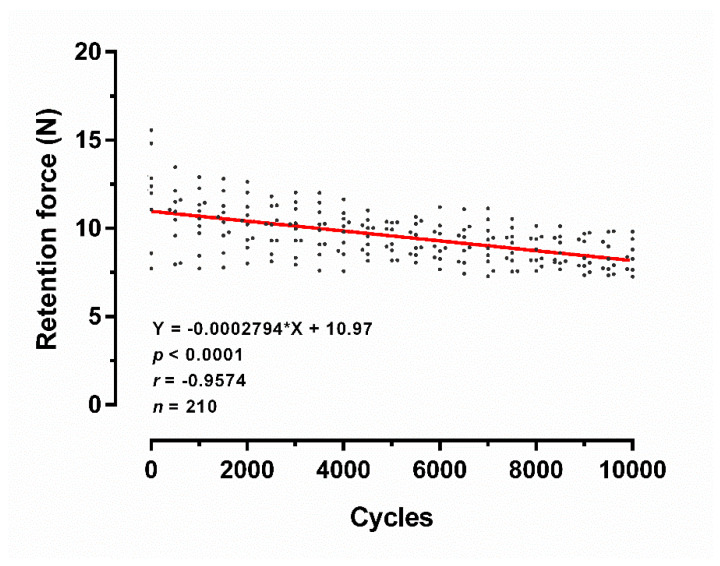
Effect of cyclic dislodgement on the retention force of the PVS attachment (SH60). Linear regression between the cycle’s times and retention force. Black dots represent each measurement at different times of cyclic dislodgement (*n* = 210).

The correlation between the cyclic dislodgement and retention force for a PEEK post-core restoration with a PVS attachment system (SH60) is shown in Figure 4. The results of Pearson correlation coefficient showed a statistically significant linear relationship between the cyclic times and retention force (*p* < 0.0001). Furthermore, there was a strong negative linear relationship between the cyclic times and retention force (*r* = −0.9574, *n* = 210).

### 3.2. Retention Force of Different Attachments before and after Aging

**Figure 5:**
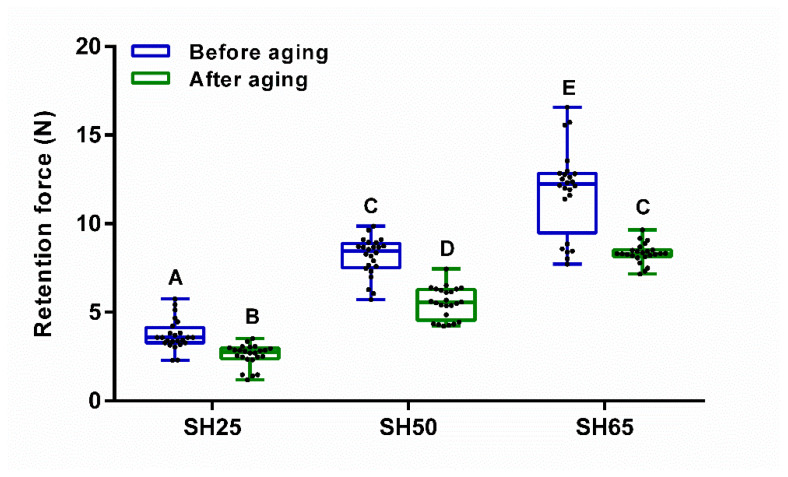
Box-and-whisker plot of retention force for the PVS attachments with three different degrees of Shore hardness (SH25, SH50, and SH65) before and after the aging treatment. Black dots represent each measurement (n = 24). Different letters indicate a statistically significant difference, determined by two-way ANOVA (Tukey’s multiple comparisons test; *p* < 0.05). The “A”, “B”, “C”, “D” and “E” letters indicate the level of statistical difference between each group.

Figure 5 shows the retention force of the PVS attachments with different degrees of Shore hardness (SH25, SH50, and SH65) before and after aging. A two-way ANOVA was conducted that examined the effect of the type of attachments and aging on the retention force. There was a statistically significant interaction (F (2, 138) = 10.95, *p* < 0.0001). Each main effect was also significant for aging (F (2, 138) = 374.7, *p* < 0.0001) and the type of PVS attachments (F (1, 138) = 137.2, *p* < 0.0001). A simple main effect analysis revealed that the retention forces before aging were significantly higher than those after aging for SH25 (*p* = 0.0198), SH50 (*p* < 0.0001) and SH65 (*p* < 0.0001), respectively. Regarding the different attachments, the retention forces of SH 65 were statistically higher than those of SH25 and SH50 (*p* < 0.0001).

## 4. Discussion

The aim of this study was to evaluate retention of a novel PEEK post-core restoration with PVS attachments. After cyclic dislodgement, the results showed a strong negative linear relationship between the cyclic times and retention force (*p* < 0.0001). Herein, the first null hypothesis was rejected. In our results, the retention forces of PVS overdenture attachment were gradually decreased, which is in accordance with previous investigations of overdenture attachments [27,28,29]. Previous studies demonstrated that clear wear and microcracks on the PVS attachments caused by cyclic dislodgment led to their retention forces decreased [22,23]. Considering most overdenture attachments, such as locator [27], bar-type [28], and stud-type attachments [29], a decrease in retention force is observed after long-term repeated dislodging. Nonetheless, the retention forces of PVS attachments (SH60) during 10,000 cycles are constantly higher than 7 N, probably sufficient to the minimum requirements (>5 N) for overdenture stability [30]. Notably, Schweyen et al. reported no significant changes in retention force with PVS attachments after 5000 cycles of repeated dislodging, which is controversial with our results [22]. This difference might be caused by the 10,000 insertion–separation cycles time and PEEK post-core restoration used in our study. Considering the clinical application, the PVS attachments were subjected to 10,000 cycles, corresponding to approximately ten years of clinical use, whereas insertion–separation under in vivo conditions are more complicated and should be considered in further researches.

The second null hypothesis, concerning the effects of the different degrees of Shore hardness and aging treatment on the retention forces, has to be rejected. Regarding the different degrees of Shore hardness, our result is in agreement with previous studies [22,23]. Meththananda et al. demonstrated the well-defined relationship between Shore hardness of the dental elastomers and Young’s modulus [31]. Schweyen et al. reported a positive correlation between the Shore hardness, as a degree of stiffness, and retention forces of PVS attachments [22,23]. Considering the specific clinical applications, different retention forces of attachments are required. For instance, it might be easier and feasible for neurologic patients affected with hand dexterity to insert and remove attachment-retained denture with low retention forces [32]. In addition, the retentive properties were significantly affected by the aging treatment. All three types of PVS attachments were aging-treated by the mechanical dislodgement in the artificial saliva. On the one hand, the effect of repeated dislodgement might reduce the hardness and stiffness of PVS and increase wear of the surface of the PEEK core, which could lead to decreasing retention forces. On the other hand, artificial saliva could affect the retention of the PEEK post-core with PVS attachments. Previous studies reported that artificial aging decreased the retention in different attachment systems, such as ball attachment [33] and locator attachment [34]. Specifically, Schweyen et al. investigated that the retention forces for Locator attachments and PVS attachments were significantly decreased by artificial saliva, regardless of the number of dental implants [23]. In contrast, Bayer et al. reported the retentive forces of ball attachments with metal inserts were increased by a physiological sodium chloride solution, which might be caused by its low viscosity [33]. In our study, the main reason for the decrease in retention force might be attributed to the fact that mucins in the artificial saliva, as a viscous layer on parts of attachment, reduce abrasion and facilitate its separation [23,33,34]. Admittedly, in the present study, the aging treatment was performed at room temperature, which cannot mimic the thermal cyclic in oral conditions. Thus, further investigations are required to evaluate the longevity of the PEEK post-core restoration with PVS attachments.

## 5. Conclusions

Within the limitations of this in vitro study, the following conclusions were drawn.

1. The PEEK post-core restoration with PVS attachments showed the favorable retention force, which corresponds to an appropriate retention force for ten years of clinical use.
2. The retention force of the three types of PVS attachments increased as its Shore hardness increased, which can expand clinical application in specific cases.
3. Retention forces of the PVS attachments were affected by the aging treatment, probably caused by the repeated wear and artificial saliva.

Therefore, we concluded that the novel PEEK post-core restoration combined with PVS attachments might be a promising concept for dental prostheses. Nevertheless, further systematic investigations are required.
